# Is survival influenced by metastatic site in synchronous metastatic pancreatic adenocarcinoma (PDAC)? A prospective real-world BACAP study

**DOI:** 10.1016/j.esmogo.2025.100144

**Published:** 2025-02-26

**Authors:** E. Alouani, C. Canivet, B. Bournet, L. Buscail, J. Selves, B. Napoleon, L. Palazzo, N. Flori, P. Guibert, A.-C. Brunac, C. Maulat, F. Muscari, F.-Z. Mokrane, S. Gourgou, L. Roca, R. Guimbaud, N. Fares, BACAP Consortium, Barbara Bournet, Cindy Canivet, Louis Buscail, Nicolas Carrere, Fabrice Muscari, Bertrand Suc, Rosine Guimbaud, Corinne Couteau, Marion Deslandres, Pascale Rivera, Anne-Pascale Laurenty, Nadim Fares, Karl Barange, Janick Selves, Anne Gomez-Brouchet, Bertrand Napoleon, Bertrand Pujol, Fabien Fumex, Jerome Desrame, Christine Lefort, Vincent Lepilliez, Rodica Gincul, Pascal Artru, Lea Clavel, Anne-Isabelle Lemaistre, Laurent Palazzo, Jerome Cros, Sarah Tubiana, Nicolas Flori, Pierre Senesse, Pierre-Emmanuel Colombo, Emmanuelle SamailScalzi, Fabienne Portales, Sophie Gourgou, Claire Honfo Ga, Carine Plassot, Julien Fraisse, Fred eric Bibeau, Marc Ychou, Pierre Guibert, Christelle de la Fouchardiere, Matthieu Sarabi, Patrice Peyrat, Severine Tabone-Eglinger, Caroline Renard, Guillaume Piessen, Stephanie Truant, Alain Saudemont, Guillaume Millet, Florence Renaud, Emmanuelle Leteurtre, Patrick Gele, Eric Assenat, Jean-Michel Fabre, Francois-Regis Souche, Marie Dupuy, Anne-Marie Gorce-Dupuy, Jeanne Ramos, Jean-Francois Seitz, Jean Hardwigsen, Emmanuelle Norguet-Monnereau, Philippe Grandval, Muriel Duluc, Dominique Figarella-Branger, Veronique Vendrely, Clement Subtil, Eric Terrebonne, Jean-Frederic Blanc, Etienne Buscail, Jean-Philippe Merlio, Dominique Farges Bancel, Jean-Marc Gornet, Daniela Geromin, Geoffroy Vanbiervliet, Anne-Claire Frin, Delphine Ouvrier, Marie-Christine SaintPaul, Philippe Berthelemy, Chelbabi Fouad, Stephane Garcia, Nathalie Lesavre, Mohamed Gasmi, Marc Barthet, Vanessa Cottet, Cyrille Delpierre

**Affiliations:** 1 The CHU and the University of Toulouse, Toulouse, France; 2 Jean Mermoz Hospital, Lyon, France; 3 Trocadero Clinic, Paris, France; 4 The Department of Pathology, Beaujon Hospital and Paris 7 University, Clichy, France; 5 The Biobank, Bichat Hospital and Paris 7 University, Paris, France; 6 The Cancer Institute and the University of Montpellier, Montpellier, France; 7 The Leon Berard Cancer centre, Lyon, France; 8 The Department of Digestive Surgery, the CHU and the University of Lille, Lille, France; 9 The CHU and the University of Montpellier, Montpellier, France; 10 La Timone Hospital and the University of Marseille, Marseille, France; 11 The CHU and the University of Bordeaux, Bordeaux, France; 12 Saint Louis Hospital and Paris 7 Diderot University, Paris, France; 13 The CHU and the University of Nice, Nice, France; 14 Pau Hospital, Pau, France; 15 The CHU Nord Hospital and the University of Marseille, Marseille, France; 16 INSERM UMR866 and the University of Dijon, Dijon, France; 17 INSERM UMR1027 and the University of Toulouse, Toulouse France; 1 Digestive Medical Oncology, Centre Hospitalier Universitaire de Toulouse—Hôpital Rangueil, Toulouse, France; 2 Department of Gastroenterology and Pancreatology, Centre Hospitalier Universitaire de Toulouse—Hôpital Rangueil, Toulouse, France; 3 Department of Pathology, Centre Hospitalier Universitaire de Toulouse, Toulouse, France; 4 Department of Gastroenterology and Pancreatology, Hôpital Privé Jean Mermoz, Lyon, France; 5 Service de gastro-entérologie, Clinique du Trocadéro, Paris, France; 6 Clinical Nutrition and Gastroenterology Department, ICM—Institut du Cancer de Montpellier, Montpellier, France; 7 Centre Léon Bérard, Endoscopy Unit, Centre Léon Bérard, Lyon, France; 8 Department of Digestive Surgery, Centre Hospitalier Universitaire de Toulouse—Hopital Rangueil, Toulouse, France; 9 Department of Radiology, Centre Hospitalier Universitaire de Toulouse—Hopital Rangueil, Toulouse, France; 10 Biostatistics unit, ICM—Institut régional du Cancer de Montpellier, Val d’Aurelle, Montpellier, France

**Keywords:** pancreatic cancer, survival, metastatic site, lung-only metastases

## Abstract

**Background:**

Advanced pancreatic ductal adenocarcinoma (PDAC) carries a dismal prognosis with a 5-year survival rate of 3%. While treated as an even population, previous retrospective studies suggested significantly different survival rates for patients with lung-only metastases when compared with other patients. This study aims to explore prospectively the difference in survival outcome based on initial site of metastases in synchronous metastatic PDAC.

**Patients and methods:**

This is a prospective observational study including all adult patients with synchronous metastatic PDAC in BACAP (national Anatomo-Clinical Database on Pancreatic Adenocarcinoma). Data regarding patients’ demographics, tumor characteristics and survival outcomes were analyzed.

**Results:**

Overall, 559 patients were included (52.8% male, mean age 69 years) of which 26 (4.7%), 65 (11.6%), 299 (53.5%) and 169 (30.2%) patients had lung-only, peritoneal-only, liver-only and multi-site metastases at diagnosis, respectively. The median overall survival (OS) was significantly different according to metastatic site (*P* < 0.001) with a median OS for lung-only, peritoneum-only, liver-only and multi-site of 12.6 months [95% confidence interval (CI) 9.7-16.9 months], 8.6 months (95% CI 5.4-11.5 months), 7.9 months (95% CI 6.5-8.9 months) and 4.5 months (95% CI 3.9-5.8 months), respectively. The median progression-free survival (PFS) was also significantly different according to metastatic site (*P* < 0.01) with a median PFS of 6.3 months (95% CI 2.7-9.1 months), 5.1 months (95% CI 3.7-6.2 months), 4.7 months (95% CI 3.3-5.7 months) and 3.2 months (95% CI 2.6-4.1 months), respectively.

**Conclusions:**

Patients with lung-only metastases represented 4.7% of synchronous metastatic PDAC patients and exhibited improved survival. These results suggest that a subset of patients with synchronous metastatic PDAC could benefit from more aggressive locoregional treatments.

## Highlights

• OS and PFS varied significantly by metastatic site in synchronous metastatic PDAC.
• Lung-only metastases represented 4.7% of synchronous metastatic PDAC.
• Patients with lung-only PDAC metastases exhibited the most favorable prognosis.
• Age >80 years old and neutrophils <7 G/l were associated with lung-only metastases.

## Introduction

Most patients with pancreatic ductal adenocarcinoma (PDAC) present with metastatic disease at the time of diagnosis, carrying a dismal prognosis with a 5-year survival rate of 3% (SEER). In the majority of patients, the liver is the predominant site of metastasis,^1^ which is often associated with poorer outcomes due to the liver’s critical role in metabolism and detoxification, further compromising the patient’s overall condition. However, a subset of patients show a change in this metastatic pattern and present distant metastases without involving the liver, raising important questions about whether these metastatic patterns influence survival.

While treated as an even population, emerging evidence suggests that the metastatic site could be a significant factor in predicting survival outcomes. In particular, lung-only metastases have been associated with improved survival rates in comparison with patients with liver-only or multi-site metastases.^2^, ^3^, ^4^, ^5^, ^6^, ^7^ For example, Zheng et al. reported on 232 patients who had a relapse following pancreatectomy that those patients with lung-only metastases had longer survival after recurrence and overall survival (OS) of 20 and 36 months, respectively, compared with 5 and 10 months in patients with liver recurrence.^7^ However, these studies primarily focused on patients who underwent resection of the primary tumor and later developed metachronous metastases. Whether this observation is solely found in this particular setting or is a general phenomenon where patients with isolated lung metastases present with a more favorable prognosis regardless of the setting (metachronous or synchronous) is unknown. Furthermore, most of the existing literature is constrained by limited number of patients, monocentric and retrospective nature of many studies, potentially limiting the robustness of the conclusions. Using data from the large, multicentric prospective real-world BACAP cohort, this study aims to determine whether the site of metastasis at diagnosis has a significant impact on survival outcomes in synchronous metastatic PDAC.

## Patients and methods

### Patients

This is a prospective observational study including all adult patients with histologically proven synchronous metastatic PDAC in the BACAP (national Anatomo-Clinical Database on Pancreatic Adenocarcinoma) database diagnosed from 2014 to 2023.^8^ ‘Synchronous metastases’ refers to metastases discovered at the time of diagnosis of the primary tumor or within the following 3 months. The patients were divided into four groups: lung-only, peritoneal-only, liver-only and multi-site metastases based on radiological assessment at diagnosis.

The metastatic organs involved were identified through imaging studies, using either a computerized tomography scan or a magnetic resonance imaging scan, and subsequently confirmed by a multidisciplinary tumor board.

Isolated metastases were defined as the presence of significant lesions confined to a single organ. Metastatic tumors affecting multiple organs were classified as multiple site metastases.

Patients with isolated metastases and ascites at diagnosis were excluded from the study because, without ascitic fluid analysis and/or exploratory laparoscopy, we could not rule out peritoneal carcinomatosis involvement. Other exclusion criteria included patients with unknown metastatic site or without any follow-up.

The BACAP cohort was approved by the following: (i) the national French committee for data processing related to health research (Comité Consultatif sur le Traitement de l’Information en matière de Recherche dans le domaine de la Santé, CCTIRS, September 2013, Folder 13.490); (ii) the National French Data Protection Authority (Commission Nationale de l’Informatique et des Libertés, CNIL, March 2014, Authorization N°913, 462) and (iii) the ethics committee (Comité de Protection des Personnes pour la recherche biomédicale Sud-ouest et Outre-Mer I, CPP, March 2014). The protocol was deposited at ClinicalTrials.gov (NCT02818829).

### Clinical data

Clinical, biological and imaging follow-up data, including sex, age at diagnosis, primary tumor location, metastatic sites and previous lines, were collected from the BACAP database managed by the Montpellier Cancer Institute Data Centre with the Clinsight® software. The cut-off date for the present analysis was 5 March 2024. All patients who were alive at the time of last follow-up were censored.

### Endpoints

The primary endpoint was OS, defined as the time from the date of diagnosis (initial biopsy) to the date of death or most recent follow-up.

Other endpoints included progression-free survival (PFS) defined as the time from the initiation of first-line chemotherapy to disease progression or death from any cause. Patients alive without progression at last follow-up were censored at this date. Progression was assessed according to RECIST, version 1.1. Objective response rate (ORR) was defined as the percentage of patients with a complete or partial response according to RECIST v1.1, and disease control rate (DCR) was defined as the proportion of patients with complete/partial response and stable disease as best response.

### Statistical analysis

Quantitative variables were described using median value and range. Qualitative variables were summarized for the entire population and by cohorts, using numbers, percentages and number of missing data. The Kruskal–Wallis test was used to compare the distribution of the quantitative variables between the four groups. Proportions were compared between the four groups using chi-square tests or Fisher’s exact test when observations were at a low limit.

PFS and OS were estimated using the Kaplan–Meier method with their respective 95% confidence intervals (CIs) and were compared using a log-rank test.

Patients with lung-only metastases were compared with those with other site of metastases.

The association between clinical baseline characteristics and patients with lung-only metastases was evaluated with univariate analysis. Parameters with a *P* value <0.2 and <20% missing data in the univariate analysis were then evaluated in the multivariable model, providing an odds ratio (OR) and its 95% CI. Backward elimination strategy was used as the selection variable procedure. Parameters with a *P* value <0.05 were considered statistically significant. All the statistical tests were bilateral with a significance level of 5% and were carried out using Stata, version 16.0 (StataCorp LLC, College Station, TX).

## Results

### Patients

**Figure 1:**
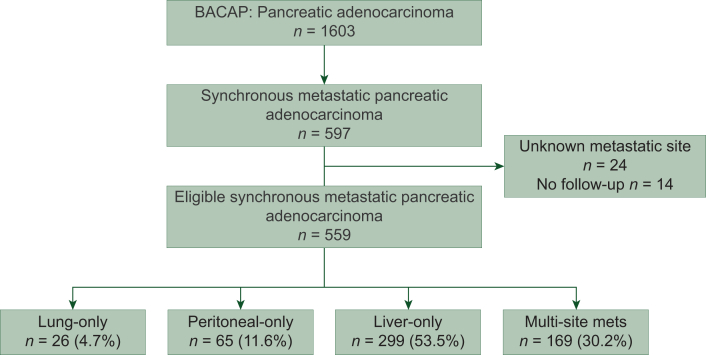
**Flow chart of patients included in the study.** BACAP, national Anatomo-Clinical Database on Pancreatic Adenocarcinoma.

Out of 1603 patients in the BACAP cohort, the study included 559 patients with synchronous metastases diagnosed between 2014 and 2023 in 15 tertiary cancer centers in France. The most common site of metastases was liver-only (*n* = 299, 53.5%), followed by multi-site (*n* = 169, 30.2%), peritoneal-only (*n* = 65, 11.6%) and lung-only (*n* = 26, 4.7%) (Figure 1). Patient and tumor baseline characteristics are reported in Table 1. Overall, the median age was 69 years (range 39-91 years) and 52.8% of patients were male; 258 patients (47.2%) had head of pancreas PDAC, 80.3% of patients had an Eastern Cooperative Oncology Group (ECOG) performance status 0 or 1. Most patients (79.6%) received at least one line of chemotherapy. The most frequent regimen administered in the first-line setting was folfirinox (52.2%) followed by gemcitabine (29%).

**Table 1:** Patient and tumor characteristics at diagnosis in patients with synchronous metastatic PDAC

	Liver	Peritoneal	Lung	Multi-site	Total	*P* value
	*n* = 299 (%)	*n* = 65 (%)	*n* = 26 (%)	*n* = 169 (%)	*N* = 559 (%)	
Median age (range), years	69.0 (36-90)	68.0 (40-87)	72.5 (55-88)	69.0 (39-91)	69.0 (36-91)	0.304
Sex ratio (male/female)	1.0 (153/146)	1.4 (38/27)	1.2 (14/12)	1.1 (90/79)	1.1 (295/264)	0.758
ECOG-PS, *n* (%)						0.706
ECOG 0	105 (39.8)	21 (39.6)	4 (16.0)	39 (25.8)	169 (34.3)	
ECOG 1	112 (42.4)	21 (39.6)	16 (64.0)	78 (51.7)	227 (46.0)	
ECOG ≥2	47 (17.8)	11 (20.8)	5 (20.0)	34 (22.5)	97 (19.7)	
Missing	35	12	1	18	66	
Alcohol, *n* (%)	105 (36.0)	15 (23.8)	7 (30.7)	59 (35.4)	186 (33.9)	0.248
Missing	7	2	0	2	11	
Tobacco smoking, *n* (%)	156 (52.5)	31 (49.2)	14 (53.8)	87 (51.7)	288 (52)	0.966
Missing	2	2	0	1	5	
Median BMI (kg/m^2^)	23.5	23.7	24.7	23.4	23.5	0.388
History of pancreatic disease^a^, *n* (%)	11 (3.7)	7 (10.8)	5 (19.2)	11 (6.5)	24 (4.3)	0.165
Primary tumor location, *n* (%)						<0.001
Head	157 (53.8)	24 (38.1)	16 (61.5)	61 (36.7)	258 (47.2)	
Body/tail	135 (46.2)	39 (61.9)	10 (38.5)	105 (63.3)	289 (52.8)	
Missing	7	2	0	3	12	
Laboratory (median value), *n* (%)						
Hemoglobinemia (g/dl)	12.9	12.8	12.6	12.4	12.8	0.297
Leucocytes (10^9^/l)	8.5	8.2	7	8.7	8.4	0.007
Neutrophils (10^9^/l)	6.6	5.6	4.0	6.6	6.3	0.060
CRP (mg/l)	18.4	14.4	6.5	30.3	18.4	<0.001
Albumin (g/l)	37.0	37.5	39.5	37.0	37.0	0.263
Bilirubin (μmol/l)	13.7	10.0	10.6	11.0	12.0	0.042
Ca 19.9 (μmol/l)	1568.5	288	178.4	2481.0	913.0	<0.001
ACE (UI/l)	13.3	4.7	2.9	16.9	11.4	<0.001

ACE, angiotensin-converting enzyme; BMI, body mass index; Ca 19.9, carbohydrate antigen 19-9; CRP, C-reactive protein; ECOG, Eastern Cooperative Oncology Group; PDAC, pancreatic ductal adenocarcinoma; PS, performance status.

a Including intraductal papillary mucinous neoplasm, mucinous cystadenoma, serous cystadenoma, chronic calcifying pancreatitis, hereditary chronic pancreatitis.

### Survival and response to treatment

**Figure 2:**
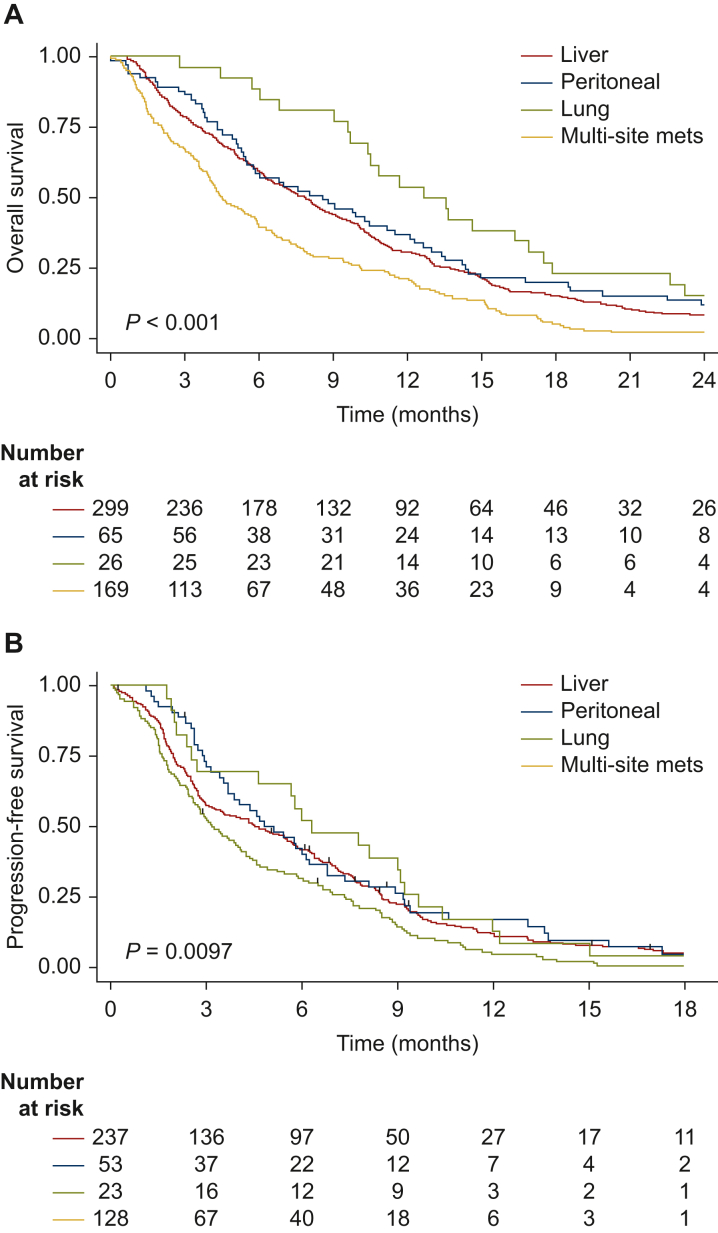
**Kaplan-Meier survival curves according to metastatic site.** Overall survival (A) and progression-free survival (B) according to metastatic site at diagnosis in synchronous metastatic pancreatic ductal adenocarcinoma.

**Figure 3:**
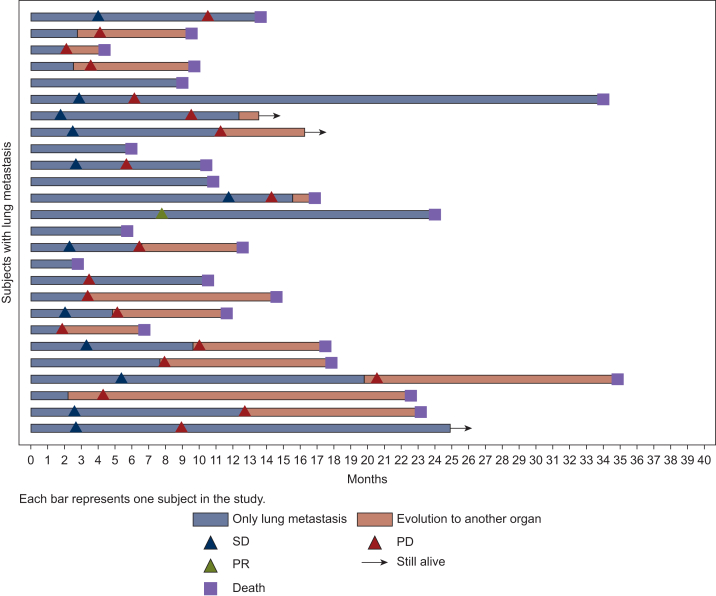
Swimmer plot showing time from diagnosis to evolution to another organ in the lung-only metastases group.

At the data cut-off date, the median follow-up was 42.1 months (95% CI 36.9-54.5 months). Overall, 320 patients (72.6%) had progressed and 513 patients (91.8%) had died. The median OS was 6.9 months (95% CI 6.0-7.8 months) (Figure 2). The median PFS was 4.3 months (95% CI 3.5-4.9 months). The median OS was significantly different according to metastatic site (*P* < 0.001) with a median OS for lung-only, peritoneum-only, liver-only and multi-site of 12.6 months (95% CI 9.7-16.9 months), 8.6 months (95% CI 5.4-11.5 months), 7.9 months (95% CI 6.5-8.9 months) and 4.5 months (95% CI 3.9-5.8 months), respectively. The median PFS was also significantly different according to metastatic site (*P* < 0.01) with a median PFS of 6.3 months (95% CI 2.7-9.1 months), 5.1 months (95% CI 3.7-6.2 months), 4.7 months (95% CI 3.3-5.7 months) and 3.2 months (95% CI 2.6-4.1 months), respectively. In addition, median time to progression to another organ was significantly different for lung-only, liver-only and peritoneum-only with a median of 9.1 months (95% CI 5.1-11.8 months), 6.3 months (95% CI 5.4-11.2 months) and 6.0 months (95% CI 4.8-9.2 months), respectively. Moreover, 8/26 (30.8%) and 3/26 (11.5%) patients had no tumor progression to another organ at 12 months and 24 months of diagnosis, respectively (Figure 3).

In total, 377 patients were assessable for tumor response according to RECIST v1.1 criteria. ORR was 19.6%, with complete responses in 1% of cases (*n* = 4) (Table 2). DCR was observed in 57.3% of patients. There was no significant difference in terms of ORR between the different groups (*P* = 0.325).

**Table 2:** Treatment and outcome

	Liver	Peritoneal	Lung	Multi-site	Total	*P* value
	*n* = 239	*n* = 53	*n* = 23	*n* = 130	*N* = 445	
First-line treatment regimen, *n* (%)						0.953
Folfirinox	126 (55.3)	30 (58.9)	10 (47.6)	55 (44.7)	221 (52.2)	
Folfox	13 (5.7)	3 (5.9)	2 (9.5)	10 (8.1)	28 (6.6)	
Folfiri	3 (1.3)	0 (0.0)	0 (0.0)	1 (0.8)	4 (0.9)	
Capecitabine	1 (0.4)	0 (0.0)	0 (0.0)	0 (0.0)	1 (0.2)	
Gemcitabine-nab-paclitaxel	16 (7.0)	2 (3.9)	1 (4.8)	11 (8.9)	30 (6.7)	
Gemcitabine	64 (28.1)	15 (29.4)	7 (30.4)	43 (35.0)	129 (29.0)	
Gemox	1 (0.4)	1 (2.0)	0 (0.0)	2 (1.6)	4 (0.9)	
Other^a^	4 (1.8)	0 (0.0)	1 (4.8)	1 (0.8)	6 (1.3)	
Missing	11	2	2	7	22	
Objective response, *n* (%)	45 (21.5%)	8 (16.7%)	1 (5.0%)	20 (20.0%)	74 (19.6%)	0.325
Best response, *n* (%)						0.159
Complete response	3 (1.4%)	0 (0.0)	0 (0.0)	1 (1.0%)	4 (1.0%)	
Partial response	42 (19.9%)	8 (16.7)	1 (5.0)	19 (18.3%)	70 (18.3%)	
Stable disease	73 (34.6)	26 (54.2)	11 (55)	32 (30.8)	142 (37.1)	
Progression disease	91 (43.1)	14 (29.2)	8 (40)	48 (46.2)	161 (42)	
Not evaluable	2 (0.9)	0 (0)	0 (0)	4 (3.8)	6 (1.6)	
Progression-free survival, median (95% CI), months	4.7 (3.3-5.7)	5.1 (3.7-6.2)	6.3 (2.7-9.1)	3.2 (2.6-4.1)	4.3 (3.5-4.9)	0.0097
Overall survival, median (95% CI), months	7.9 (6.5-8.9)	8.6 (5.4-11.5)	12.6 (9.7-16.9)	4.5 (3.9-5.8)	(6.0-7.8)	<0.001
Time to progression to another organ, Median (95% CI), months	6.34 (5.35-7.22)	6.30 (4.82-9.19)	9.10 (5.15-11.79)	4.10 (3.51-5.19)	5.74 (5.15-6.40)	<0.001

CI, confidence interval.

a Refers to patients in clinical trials evaluating investigational drugs.

### Markers associated with lung-only metastases

Baseline characteristics at diagnosis (age, sex, tobacco and alcohol consumption, body mass index, ECOG, history of underlying pancreatic disease, primary tumor location, histological type and all biological parameters) were tested in univariate analysis to identify markers associated with the presence of lung-only metastases. Overall, age >80 years old, history of underlying pancreatic disease, neutrophils <7 giga/l, C-reactive protein <10, albumin >35, carbohydrate antigen 19-9 <200 μmol/l and angiotensin-converting enzyme <6 ng/l were statistically significant. The last three biological criteria were not included in multivariate model due to missing data, for statistical relevance.

In multivariate analysis (*n* = 506), the results showed that age >80 years and neutrophils <7 giga/l were independently associated with lung-only metastases (Table 3).

**Table 3:** Markers associated with lung-only metastases (multivariate analysis) (*n* = 506)

	Effect	Univariate logistic model				Multivariate logistic model			
		OR	95% CI		*P* value	OR	95% CI		*P* value
Age, years (*n* = 559)					0.014				0.014
	**≤80**	1							
	>80	3.534	1.329	8.679		3.603	1.402	9.261	
History of pancreatic disease^a^ (*n* = 559)					0.091				
	**No**	1							
	Yes	2.405	0.868	6.667					
Primary tumor location (*n* = 547)					0.132				
	**Head**	1							
	Other	0.542	0.241	1.216					
Neutrophils (10^9^/l) (*n* = 506)					0.001				0.001
	**<7**	1				1			
	≥7	0.171	0.050	0.580		0.175	0.051	0.596	
CRP (mg/l) (*n* = 346)					0. 012				
	**<10**	1							
	≥10	0.295	0.111	0.783					
Albumin (g/l) (*n* = 312)					0.097				
	**<35**								
	≥35	3.179	.684	14.767					
Ca 19.9 (μmol/l) (*n* = 387)					0.084				
	**<200**	1							
	≥200	0.421	0.158	1.119					
ACE (UI/l) (*n* = 331)					0.046				
	**<6**	1							
	≥6	0.346	0.120	0.997					

Sex, ECOG-PS, alcohol, smoking status, BMI, hemoglobin and bilirubin were not statistically significant in the univariate analysis.

Bold in table are the references used for univariate and multivariate analyses.

ACE, angiotensin-converting enzyme; BMI, body mass index; Ca 19.9, carbohydrate antigen 19-9; CI, confidence interval; ECOG, Eastern Cooperative Oncology Group; OR, odds ratio; PS, performance status.

a Including intraductal papillary mucinous neoplasm, mucinous cystadenoma, serous cystadenoma, chronic calcifying pancreatitis, hereditary chronic pancreatitis.

## Discussion

PDAC with distant metastases not involving the liver are rare tumors, particularly in the synchronous metastatic setting. Given their low incidence, the number of studies is limited and the prevalence, risk factors and survival impact of the metastatic site is not well known. Our cohort of 599 patients with synchronous metastatic PDAC is one of the largest multicenter cohorts reported to date, with the specificity of having data from the real-world setting collected prospectively. A prior retrospective study focusing on patients treated with palliative chemotherapy for stage IV PDAC in Taiwan showed that isolated lung metastases were a better prognosticator for OS in univariate and multivariate analysis.^9^ Similarly, Oweira et al. showed better outcomes for patients with isolated lung metastases compared with isolated liver metastases in stage IV PDAC within the Surveillance, Epidemiology and End Results (SEER) database.^10^ It is worth noting that the study focused on patients diagnosed between 2010 and 2013 and the SEER database lacks information regarding peritoneal metastases. This is, to the best of our knowledge, the first study evaluating prospectively the impact of metastatic site on survival in patients with synchronous metastatic PDAC in a contemporary western population. We identified 4.7% of lung-only metastases in patients with synchronous metastatic PDAC which concurred with previous studies.^9^^,^^11^ This study confirms that survival in synchronous metastatic PDAC differs based on the initial site of metastases. Specifically, patients with lung-only metastases at diagnosis almost tripled their survival compared with multi-site metastases with a median OS of 12.6 months. Furthermore, patients with isolated lung metastases had a significantly increased time to progression to another organ compared with other patients. Importantly, after 1 year since diagnosis, 30.8% of these patients still presented with isolated lung metastases. Tissera et al. showed that patients with oligometastatic lung disease (OMLD) (less than four lesions) had a longer median OS (35.7 months) compared with non-OMLD patients (26.2 months) after PDAC resection (hazard ratio 0.34, 95% CI 0.12-0.96).^12^ The number of metastatic lesions was not available and therefore could not be assessed in our study.

Conversely, there was no difference in ORR suggesting that better survival is not owing to better chemosensitivity but rather to a more indolent nature of PDAC with lung-only metastases. These observations may potentially impact the standard treatment approach of metastatic PDAC. For example, those patients may be subject to less intensive systemic treatments, such as single- or doublet-agent therapy in contrast to triplet therapy. Moreover, successful surgical intervention in patients with isolated lung recurrence of PDAC has been associated with improved survival outcomes.^13^, ^14^, ^15^, ^16^, ^17^, ^18^, ^19^, ^20^ For example, a nationwide multicenter analysis conducted in Japan found that patients who underwent pulmonary resection had significantly better survival compared with those who did not (29.2 months from recurrence versus 15.2 months, *P* < 0.001).^15^ Similarly, Breton et al. evaluated the outcomes of locoregional treatment for PDAC metastases (including different sites) in 52 patients.^20^ After a 3.7-year follow-up, the authors reported a median OS of 36.5 months following local treatment, with a significant improved survival advantage for lung-only metastases compared with liver-only metastases (not reached- versus 28 months, respectively, *P* = 0.01). Of note, the majority of these studies included patients with metachronous metastases, and there is limited data on the use of locoregional treatment for metastases in the synchronous setting. In our study, there was no information available regarding locoregional treatment of metastases. In France, as per international guidelines, locoregional treatment for metastases in PDAC is not considered standard practice.^21^ Furthermore, none of the patients in our cohort were included in clinical trials. Consequently, we expect that only a small subset of patients received such treatment. Our study observed a survival advantage for lung-only metastases in the synchronous setting. Based on this finding, we suggest that selected patients may benefit from more aggressive locoregional treatment, even in the context of synchronous metastases, as this approach could potentially further improve survival. This hypothesis warrants validation in prospective clinical trials. In addition, García-Mulero et al. found that lung metastases regardless of primary tumor origin showed a distinct inflammatory phenotype with higher levels of immune infiltrate, overexpression of programmed death-ligand 1 and cytotoxic T-lymphocyte associated protein 4 (CTLA4) pathways and up-regulation of genes predicting clinical response to programmed cell death protein 1 blockade.^22^ The latter needs to be confirmed in PDAC patients and if so, this suggests that immunotherapy warrants further investigation in lung-only metastatic PDAC. Overall, lung-only metastatic PDAC may also need to be taken into consideration for stratification in clinical trials and should be further investigated in prospective analyses to confirm the observations and determine the mechanisms that may explain the isolated occurrence of these metastases, and their better prognosis.

In our study, we found that age >80 years old and neutrophils <7 giga/l were independently associated with lung-only PDAC metastases. Age is classically a negative prognostic factor associated with PDAC^23^, ^24^, ^25^ also because older patients are diagnosed at a later stage and tend to have more comorbidities and to receive less aggressive treatment than younger patients. Conversely, our study found that age >80 years was associated with lung-only metastases and therefore a more indolent tumor nature. High neutrophil rate has conversely been clearly associated with worse prognosis in PDAC.^26^ Neutrophils inhibit the immune response by lymphocytes, natural killer cells or activated T cells (el-hag) and may therefore also contribute to a different immune microenvironment. There are other markers that have been identified in the literature associated with lung-only metastases. Several studies found that female sex was a risk factor for lung-only metastases.^9^^,^^27^, ^28^, ^29^ This was not found in our study. Interestingly, the proportion of patients with a history of pancreatic disease was enriched in the lung-only group although it did not appear as an independent marker in the multivariate analysis, possibly because of a lack of statistical power. These patients mostly present with underlying intraductal papillary mucinous neoplasm (IPMN). While IPMN-associated PDAC accounts for a small proportion of all PDAC, De la Fuente et al. showed that these patients had better survival.^30^

Following this observation, it will be intriguing to investigate specific biologic pathways that could determine each pattern of metastases. PDAC driver mutations, such as *KRAS*, *TP53*, *SMAD4* and *CDKN2A*, have been associated with a worse prognosis.^31^ A retrospective study of a limited number of patients showed the absence of the *CDKN2A* and *SMAD4* genes and their possible association with favorable prognosis in patients with lung-only relapse.^32^ Molecular analysis was not conducted in our study. However, Tissera et al.^12^ found no difference in terms of classical PDAC mutations between OMLD and non-OMLD patients. As one possible mechanism for organotropism in PDAC, overexpression of *HER2* has been described where the authors found a preponderance of *HER2* overexpression in patients with lung without liver metastases.^33^ Furthermore, preclinical evidence suggests that genetic alterations in DNA repair could be related to metastatic organotropism to the lung but this remains to be confirmed in clinic.^34^

While our study has several strengths, there were also several limitations. Firstly, our study accounts for missing data of classical prognosis factors such as *RAS* mutations which were not recorded when the data collection started and therefore could not be assessed. Secondly, inherent to the rarity of lung-only metastases, our study probably lacked the power to identify certain markers associated with lung-only metastases. Also, the incidence of lung metastases might be overestimated in our study group because some may be misread as metastatic lesions, without pathological proof but is in line with previous reports. Nevertheless, this study represents the first contemporary large-scale study in the western population providing insights into lung-only synchronous metastatic PDAC that may help physicians manage this particular group of patients.

## Conclusion

Metastatic site impacted OS and PFS in synchronous metastatic PDAC, of which lung-only metastases represented 4.7% and exhibited the most favorable prognosis of improved survival. These results suggest that a subset of patients with synchronous metastatic PDAC could benefit from more aggressive locoregional treatments and that the identification of lung-only metastases could thus be a key element in developing efficient, tailored treatment approaches in future clinical trials.
